# Depressive symptoms among residents of south Wollo zone in Northern Ethiopia after the liberation of invasion of TPLF led force

**DOI:** 10.1016/j.heliyon.2023.e13600

**Published:** 2023-02-10

**Authors:** Seid Ali Tareke, Mesfin Esayas Lelisho, Abdu Hailu Shibeshi, Mohamednur Qadire Muze, Yasin Negash Jabir, Kibrealem Sisay Wolde, Fikadu Zawdie Chere, Ebrahim Chaine Gidelew, Shukure Uomer Salo, Husien Adem Argaw

**Affiliations:** a Department of Statistics, College of Natural and Computational Science, Mizan-Tepi University, Tepi, Ethiopia; b Department of Statistics, College of Natural and Computational Science, Semera University, Semera, Ethiopia; c Department of Banking and Finance, College of Business and Economics, Jimma University, Jimma, Ethiopia; d Department of Statistics, College of Natural Science, Jimma University, Jimma, Ethiopia; e Department of Accounting, College of Business and Economics, Mekdela Amba University, Mekaneselam, Ethiopia; f Department of Statistics, College of Natural and Computational Science, Werabe University, Werabe, Ethiopia; g Department of Accounting, College of Business and Economics, Mizan-Tepi University, Mizan, Ethiopia

**Keywords:** Depressive symptoms, TPLF-Led force invasion, Mental health, South Wollo

## Abstract

**Background:**

Depressive symptom is the most widely reported mental health consequence of natural or man-made disasters and traumatic events. Research on depressive symptoms in low-income nations is still scarce, although it can be a public health burden in post-conflict situations. Therefore, the primary objective of this study was to identify the prevalence and contributing factors of depressive symptoms among people of south Wollo zones following liberation from TPLF-led army invasions.

**Methods:**

A community-based cross-sectional study was conducted on South Wollo zone residents after the liberation of invasions of the TPLF-led force, from May 1st to June 1st, 2022. A self-administered questionnaire was used to collect data from residents chosen using a simple random sampling technique. This study used both descriptive and inferential analysis. To investigate the relationship between response and predictor variables, the chi-squared test of association was performed. The logistic regression was performed to identify predictors of depressive symptoms among residents.

**Results:**

The overall prevalence of depressive symptoms among residents was 51.0% [95% C.I: 48.01, 53.99]. Being female [AOR = 1.428, 95% C.I: 1.044–1.955], being an alcohol consumer [AOR = 2.051, 95% C.I: 1.349–3.119], chewing Khat [AOR = 4.617, 95% C.I: 2.873–7.418], history of mental illness [AOR = 4.316, 95% C.I: 1.263–14.751], destruction of personal property [AOR = 2.909, 95% C.I: 2.028–4.175], lacked basic needs such as food and water [AOR = 2.738, 95% C.I: 1.922–3.900], and illness without medical care [AOR = 2.369, 95% C.I: 1.684–3.331] were all found to be statistically significant at the 5% level of significance in a multivariable binary logistic regression.

**Conclusions:**

The main finding of this study discovered that residents had a higher prevalence of depressive symptoms following liberation from TPLF-led army invasions. Being an alcoholic, chewing Khat, having a previous history of mental illness, destruction of personal property, lack of food or water, and illness without medical care were associated with an increased risk of developing depressive symptoms. Interventions based on influencing factors should be performed to ensure residents' mental health.

## 1 Introduction

Since June 2021, the TPLF forces have been waging an open war against Ethiopia's Amhara and Afar regions, causing great economic and social hardship for defenseless inhabitants. The invading force targeted several rural farmers, city dwellers, teachers, and medical professionals in the South Wollo Zones of the Amhara region who were not politically active [1]. In addition, the invaders destroyed public facilities like hospitals, schools, TVET colleges, and financial and banking sectors, as well as looted and ruined private assets on purpose [1]. It has led to the destruction of civilian infrastructure, the internal displacement of millions of civilians, financial and family losses, and the disruption of the culture and values of millions of people living in these places. Furthermore, the conflict has resulted in 1035 unplanned pregnancies, many cases of sexual gender-based violence, rape and gang rape, and other forms of physical and sexual abuse of women [2]. Furthermore, armed conflicts are linked to poverty, unemployment, communal violence, unstable living conditions, and changes in the social dynamic. This makes the post-war scenario strongly linked to a lower quality of life, which leads to different mental health issues [3].

Depression is defined as a common mental illness that is characterized by persistent sadness, loss of interest in typically enjoyable activities, difficulty performing daily tasks, and other symptoms [4]. Additionally, the condition can affect one's ability to think, remember, and sleep [5]. This is a significant, recurring disorder that reduces one's quality of life and ability to fulfill social and familial obligations [6]. It contributes significantly to the burden of disease on the planet and is a leading cause of disability [7].

Mental illness is recognized as a major public health concern of a conflict-affected population [[8], [9], [10]]. Any traumatic event experienced during combat increases the risk of mental health issues such as post-traumatic stress disorder (PTSD), anxiety, and depression symptoms as well as worse life outcomes [3,11,12]. Being female, having a personal history of mental illness, being unemployed after the event, having persistent physical problems, and having damage to property such as a house were all risk factors for depressive symptoms [[13], [14], [15], [16]]. Pre-existing factors such as substance addiction history, and post-trauma elements such as lack of food or water, injury, and loss of family member/friend have all been identified as contributing to the development of depressive symptoms [17]. Tareke et al. found that having trouble sleeping, drinking alcohol, chewing Khat, smoking, and being a woman were all significantly associated with depressive symptoms [18]. Decreased social interaction, modifications to daily routines, and shifts in norms were all found to be factors in depressive symptoms by Brooks et al. [19].

Mental health conditions like depressive symptoms are major repercussions of armed conflict that can have both short-term effect and long-term effects on those who reside in conflict-affected areas [20,21]. Depression causes passivity, disappointment, and interpersonal, professional, and social relationship problems [22]. Because of this, it is vital to examine the prevalence and related aspects of depressive symptoms, yet doing so is uncommon in many developing nations, such as Ethiopia.

In general, this study addressed the following basic research questions: (i) What is the prevalence of depressive symptoms in the study area as a result of the invasion of a TPLF-led force? (ii) Which factors are statistically associated with depressive symptoms among study participants? Therefore, the main objective of this study was to identify the prevalence and contributing factors of depressive symptoms among people of the south Wollo zones following liberation from TPLF-led army invasions. Unlike most previous studies, this one sought to address depressive symptoms caused by conflict in the general population. The study's findings will assist health professionals, non-governmental organizations, and psychological centers in developing appropriate plans and interventions to provide evidence-based treatment for patients with depressive symptoms. Furthermore, it can also be used as a baseline for those who want to research related conditions in this area.

## 2 Material and methods

### 2.1 Study design and setting

A community-based cross-sectional study was undertaken on south Wollo zone people after liberation from TPLF-led military invasions from May 1st to June 1st, 2022. South Wollo is a zone in the Amhara region of Ethiopia. It has a population density of 147.58 people per square kilometer and covers an area of 17,067.45 square kilometers. Pretested and face-to-face questionnaires interview were used to collect data from residents selected using a simple random sample procedure. Those who were unable to provide information were removed from the study.

### 2.2 Inclusion and exclusion criteria

Those who have lived in the study area for at least six years and are aged 15 years and above were considered for this study. Participants who were unable to provide information were excluded from the study.

### 2.3 Data collection tools and measurements

Structured and pretested questions delivered by interviewers were used to collect data. It was obtained by four BSc nurses under the supervision of one psychiatry professional regularly. The survey's design was translated from English to Amharic and back to English to maintain uniformity. Data collectors received training on how to conduct participant interviews, clarify unclear questions, and describe the study's objectives.

### 2.4 Sampling method

Using a single population proportion formula and a simple random sampling method, the sample size was determined. A 37.7% prevalence of depressive symptoms among residents [18], along with a 3% margin of error and a 10% non-response rate, were used to estimate the number of samples needed for the study.

(1) $$n=\frac{{\left(Z_{\frac{\alpha }{2}}*\left(p\left(1-p\right)\right)\right)}^{2}}{d^{2}}=\frac{1.96^{2}*0.377*\left(1-0.377\right)}{0.03^{2}}=1003$$

Then the sample size for this study using equation (1) becomes 1003 + 101 = 1104. However, 29 questionnaires were excluded as a result of incomplete information, and finally, 1075 subjects participated.

### 2.5 Tools of data collection

The Center for Epidemiologic Studies Depression scale (CES-D) questionnaire was used to evaluate depression symptoms (CES-D) [23]. This scale has 20 items that describe the frequency of feelings experienced by subjects in the previous week. Each of the items has 4 options ranging from 0 to 3, that is (rarely, <1 day) to 3 (frequently or always, 5–7 days). The scoring of positive items is reversed. The overall score goes from 0 to 60, with a higher number suggesting more severe symptoms of depression. Individuals attaining a total score of 16 or more were assessed to have depression symptoms [24]. We checked internal consistency and reliability in this study, and thus Cronbach's alpha was 0.874.

### 2.6 Variables in the study

The dependent variable was depressive symptoms, defined as follows:

$$Yi=\left\{ \begin{array}{c} 1,Yes\;\left(presence\;of\;depressive\;symptoms\right)\;\\ 0,No\;\left(absence\;of\;depressive\;symptoms\right)\; \end{array}\right.$$

Independent variables were:

Demographic variables (gender, age (in years), marital status, occupation, and education level). Substance-related factors (consumption of alcohol, cigarette smoking, and Khat chewing), War related and clinical variables (previous history of mental illness, destruction of personal property, lack of food and water, injury, and illness without medical care).

### 2.7 Operational definition

• History of mental illness: Respondents were questioned about their past experiences with mental illness by asking them if they had ever received a diagnosis.
• Personal property destruction refers to any damage or destruction of real or tangible personal property caused by the war that resulted from the invasion of the TPLF-led force. To examine the destruction of personal property, respondents were asked: ‘Did you lose personal property as a result of the war?.’
• The term “lack of food and water” refers to food and water shortages caused by the war that resulted from the invasion of the TPLF-led force.
• Injury refers to any type of physical injury caused by the war that resulted from the invasion of the TPLF-led force.
• Illness without medical care refers to any type of illness that a person has experienced and is unable to receive medical treatment because of the war.

### 2.8 Statistical data analysis

All of our statistical analysis was done with (IBM) SPSS version 25. We used percentages and frequency distribution to emphasize the descriptive results. To determine how the dependent and the independent variables are related, we used the chi-squared test of association. We utilized a logistic regression model to find factors linked to depression symptoms. By including all significant factors from the univariate analysis with a significance level of 25%, multivariable logistic regressions were performed [25].

### 2.9 Binary logistic regression

Binary logistic regression is used when the response variable is dichotomous and the independent variables can be of any kind. The probability P(Y=1)=π and P(Y=0)=1−π, for which E(Y)=π, are specified by the Bernoulli distribution for the Bernoulli trial.

The following describes the binary logistic regression's general model [18]:

(2) $$\log it\left(\pi \left(xi\right)\right)=\log\left(\frac{\pi \left(xi\right)}{1-\pi \left(xi\right)}\right)=\beta o+\beta_{1}X_{1}+\beta_{2}X_{2}+\ldots .+\beta_{k}X_{k}$$

Where: in equation (2); $x_{i}$ is represent the ith predictor variables in the model, π: denotes the probability of success, while 1−π: is the probability of failure, $\beta_{o}$: is the intercept term, and $\beta_{i}$: denotes the ith coefficient of the ith predictor variables in the model

### 2.10 Evaluation of model adequacy

For logistic regression, particularly for risk prediction models, the Hosmer-Lemeshow test (HL test) measures how well the model fits the data. The test determines whether or not the observed event rates in different segments of the model population are consistent with the expected event rate [26].

The Hosmer Lemeshow test statistics [27] can be defined in equation (3) as follows:

(3) $$C^{2}=\sum_{i=1}^{p}\left[\frac{{\left(O_{i}-E_{i}\right)}^{2}}{m_{i}p_{i}\left(1-p_{i}\right)}\right]$$

Where $O_{i},E_{i},m_{i,}p_{i}$ are denotes the observed events, expected, observations and, the average predicted risk for the $i^{th}$ risk desire group respectively.

The statistical hypothesis of the Hosmer Lemeshow test

H1 The model fits the data well.

H0 The model does not fit the data well.

For large p-value, meaning that if the p-value exceeds 0.05, the null hypothesis is not rejected.

## 3 Results

The study aimed to determine the prevalence and associated factors of depressive symptoms among south Wollo zone residents after the liberation of the invasion of the TPLF Led Force. Of the 1075 study participants, 549 (51.0%) had depressive symptoms, whereas 526 (49.0%) did not.

### 3.1 Demographic characteristics of the participants

Of 1075 participants who took part in this study, the majority 608 (56.6%) were males, of which 288 (47.3%) developed depressive symptoms. More than half of the respondents 535 (49.8%) were aged 31–45 years of which 280 (52.3%) experienced depressive symptoms. More than half of the respondents 656 (61.0%) were married and the majority of the respondents 570 (53.0%) were farmers. Nearly half of the respondents 533 (49.6%) had primary\secondary school education levels (Table 1).

**Table 1:** The demographic characteristics of the participants.

Variables	Category	Count N (%)	Depressive symptoms		P-value^a^
			Yes N (%)	No N (%)	
Gender of the participant	Female	467 (43.4)	261 (55.9)	206 (44.1)	0.04
	Male	608 (56.6)	288 (47.3)	320 (52.7)	
Age of the participant	15–30	411 (38.2)	219 (53.2)	192 (46.8)	0.31
	31–45	535 (49.8)	280 (52.3)	255 (47.7)	
	>45	129 (12.0)	50 (38.7)	79 (61.3)	
Marital status	Single	261 (24.3)	141 (54.0)	121 (46.0)	0.29
	Married	656 (61.0)	314 (47.8)	342 (52.2)	
	Divorced	113 (10.5)	70 (61.9)	43 (38.1)	
	Windowed	45 (4.2)	23 (51.1)	22 (48.9)	
Education level	Illiterate	189 (17.6)	97 (51.3)	92 (48.7)	0.26
	Primary\Secondary school	533 (49.6)	253 (47.4)	280 (52.6)	
	Diploma and above	352 (32.7)	197 (56.0)	155 (44.0)	
Occupation	Student	249 (23.2)	135 (54.2)	114 (45.8)	0.27
	Farmer	570 (53.0)	276 (48.4)	294 (51.6)	
	Government Employed	159 (14.8)	74 (46.5)	85 (53.5)	
	Merchant	97 (9.0)	62 (63.9)	35 (36.1)	

a Pearson chi-square p-value.

### 3.2 Substance use, clinical, and war-related events of respondents

Regarding substance usage, 206(19.2%), 238(22.1%), and 291 (27.1%) respondents were alcohol users, smokers, and Khat chewers, respectively, with 138(66.8%), 165(69.3%), and 256 (87.9%) experiencing depressive symptoms. In terms of war-related occurrences, 396 (36.8%) of respondents reported damage to personal property. According to our data, 404 (37.6%) of respondents did not have enough food or drink, and 392 (36.5%) were ill without medical attention (Table 2).

**Table 2:** Substance use, clinical, and war-related events of respondents.

Variables	Category	Count N (%)	Depressive symptoms		P-value^a^
			Yes N (%)	No N (%)	
Khat chewing	Yes	291 (27.1)	256 (87.9)	35 (12.1)	0.01
	No	784 (72.9)	292 (37.3)	492 (62.7)	
Alcohol consumption	Yes	206 (19.2)	138 (66.8)	68 (33.2)	0.01
	No	869 (80.8)	410 (47.2)	459 (52.8)	
Cigarette smoking	Yes	238 (22.1)	165 (69.3)	73 (30.7)	0.01
	No	837 (77.9)	383 (45.8)	454 (54.2)	
Destruction of personal property	Yes	396 (36.8)	309 (78.0)	87 (22.0)	0.02
	No	679 (63.2)	239 (35.2)	440 (64.8)	
Lack of food or water	Yes	404 (37.6)	310 (76.8)	94 (23.2)	0.03
	No	671 (62.4)	238 (35.4)	433 (64.6)	
Lower to higher injury	Yes	199 (18.5)	138 (69.4)	61 (30.6)	0.05
	No	876 (81.5)	410 (46.8)	466 (53.2)	
Previous history of mental illness	Yes	101 (9.4)	97 (95.7)	4 (4.3)	0.03
	No	974 (90.6)	451 (46.3)	523 (53.7)	
Illness without medical care	Yes	392 (36.5)	274 (69.9)	118 (30.1)	0.01
	No	683 (63.5)	274 (40.1)	409 (59.9)	

a Pearson chi-square p-value.

### 3.3 Univariable analysis

Covariates having a p-value of smaller than 25% were evaluated for multivariable analysis in the Univariable analysis. From the Univariable analysis, we observed that the covariate gender, previous history of mental illness, destruction of personal property, lack of food or water, ill without medical care, alcohol consumption habit, chewing Khat, and smoking habit, were found to be significantly related to depressive symptoms. However, age, marital status, occupation, and education level were not significant. Based on this conclusion, it would be best to disregard this covariate. As a result, the impacts of these significant factors should be understood more effectively using multivariable analysis.

### 3.4 Multivariable analysis

In a multivariable binary logistic regression, gender, destruction of personal property, lack of food and water, previous history of mental illness, illness without medical care, injury, chewing Khat, and alcohol consumption habit were all found to be statistically significant at the 5% level of significance (Table 2).

Depressive symptoms were 1.428 [95% CI: 1.044–1.955] times more prevalent in women than in men. The odds of having depressive symptoms were 2.051 [95% CI: 1.349–3.119] higher among drinkers than among non-drinkers. Khat chewers were 4.617 [95% CI: 2.873–7.418] times more prevalent than their counterparts to have depressive symptoms (Table 3).

**Table 3:** Multivariable Binary logistic regression result for depressive symptoms among south Wollo zone residents after the liberation of TPLF-led force, Northern Ethiopia.

Variables	Category	B	S.E.	Sig.	Exp(B)	95% C.I.for EXP(B)	
						Lower	Upper
Gender of the participant (Ref: Male)	Female	.357	.160	.026	1.428	1.044	1.955
Alcohol consumption (Ref: No)	Yes	.719	.214	.001	2.051	1.349	3.119
Cigarette smoking (Ref: No)	Yes	−.061	.229	.790	.941	.600	1.475
Khat chewing (Ref: No)	Yes	1.530	.242	<0.001	4.617	2.873	7.418
Previous history of mental illness (Ref: No)	Yes	1.462	.627	.020	4.316	1.263	14.751
Destruction of personal property (Ref: No)	Yes	1.068	.184	<0.001	2.909	2.028	4.175
Lack of food or water (Ref: No)	Yes	1.007	.181	<0.001	2.738	1.922	3.900
Injury (Ref: No)	Yes	.600	.224	.007	1.823	1.176	2.827
Illness without medical care (Ref: No)	Yes	.862	.174	<0.001	2.369	1.684	3.331

*B: Coefficient, Sig.: p-value, EXP (B): Adjusted Odds Ratio, S.E.: Standard error, ref: reference category, C.I.: 95% confidence interval for adjusted odds ratio.

According to our findings, destroying personal property is associated with depressive symptoms. Those who had their personal belongings destroyed were 2.909 [95% CI: 2.028–4.175] times more likely to have depressive symptoms than those who had not. Individuals who had a history of mental illness had a 4.316 [95% CI: 1.263–14.751] times higher prevalence of depressive symptoms than those who did not have such a history.

Those who lacked basic needs such as food and water were 2.738 [95% CI: 1.922–3.900] times more likely to suffer depressive symptoms than those who did not. Those who experienced illness without getting medical care had a 2.369 [95% CI: 1.684–3.331] greater likelihood of acquiring depressive symptoms than their counterparts. Furthermore, the risks of acquiring depressive symptoms were 1.823 [95% CI: 1.176–2.827] times greater among individuals who experienced injury compared to those who did not.

### 3.5 Evaluation Model's adequacy

The test statistic of Hosmer and Lemeshow is large (p-value = 0.252). This suggested that the model matched the data well. Furthermore, Nagelkerke's R square equals 0.573 demonstrated that the model's existing explanatory variables explained 57.3% of the variation across response variables (Table 2).

## 4 Discussion

This study aimed to assess the prevalence and associated variables of depression symptoms among people of the south Wollo zone following the liberation of the TPLF-led force's invasion. Understanding the components of depressive symptoms may aid in the creation of specific depression interventions for the general population. This is one of the few studies in developing countries that use standardized evaluation instruments and rigorous analyses. Furthermore, this is the first study on depressive symptoms among residents in the South Wollo zone after the TPLF-led force's assault was liberated. Furthermore, it adds to the evidence of the war's effects on mental health. In the current study, personal property destruction, a lack of basic needs such as food and water, being ill without medical care, alcohol usage, injury, chewing Khat, and a history of mental illness were all identified as determinant factors for depressive symptoms.

In this study, depressive symptoms were present in 51.0% of the study participants. This is consistent with prior research on Syrian refugees conducted in Turkey (58.8%) and Iran (52.1%) [28]. This figure, however, is lower than that of Syrian internally displaced individuals (70.5%). This disparity could be attributed to the length of time they have been exposed to these heinous acts. Residents in our study were subjected to these worse conditions for a shorter time than Syrians. Furthermore, our study includes all residents, not just those in refugee camps.

Women have a considerably higher prevalence of symptoms of depression, anxiety, and PTSD than men, which is unsurprising considering the restrictions enforced on women, particularly during the war regime [29]. In line with this, gender was substantially connected with a greater likelihood of depression symptoms among south Wollo zone residents following the liberation of the TPLF Led Force assault. According to the current study's findings, males were less likely to experience depressive symptoms than females. This is consistent with the findings of [30], who discovered that being a man decreased the likelihood of depression symptoms as compared to female peers [30]. One possible explanation is that women in war zones are usually subjected to harsh social control since they are expected to follow conventional patterns and demonstrate devotion to old conventions that may not correspond with their current status and wants. Furthermore, women may be forced to adapt to new roles that may raise the likelihood of acquiring depressive symptoms.

According to our study findings, drinking alcohol and chewing Khat were strongly associated with depression symptoms. People who drink alcohol are more likely to develop depressive symptoms. This is similar to previous studies [31,32]. Alcohol consumption frequently precedes the symptoms of weariness and social difficulties that lead to depression [33]. Our results also showed that Khat chewers were 4.617 times more likely to develop depressive symptoms than their peers. This could be due to the fact that when people do not have access to such substances, they are more likely to feel tired, lack energy, irritable, restless, and other unpleasant emotions, which can lead to depression symptoms.

Both economic and public health losses from this war were considerable. This can have substantial and long-term effects in terms of physical and mental health results. According to our findings, destroying personal property is connected with depression symptoms. Those who had their personal items destroyed were 2.909 times more likely to exhibit depression symptoms than those who did not. This could be due to participants' belief that such losses will be difficult or impossible to replace, making it difficult to avoid reminders of the incident, feeling self-isolated, losing hope, and being prone to stress-filled situations, all of which lead to depressive symptoms.

Those who lacked necessities like food and water were 2.738 times higher to develop depression symptoms than those who did not. This could be because household food insecurity is a major contributor to poor nutritional status among children during the war, and it is linked to the risk of stunting and being underweight in children [34]. This has direct negative implications in terms of disease, as well as the financial potential for individuals and groups, and contributes to the development of mental health issues in communities [35].

In war-torn and post-war nations, quality healthcare is rarely accessible. Residents who do not receive the proper medical attention may endure years of suffering from health issues [36,37]. Residents of affected zones in Ethiopia have lost access to crucial health services as a result of the war. Women were not given access to family planning services, prenatal and postnatal care, and were faced with unexpected pregnancies and unsafe abortions [2]. In line with this, our research discovered that those who became ill but did not gate medical assistance had a higher likelihood of developing depressive symptoms than their peers.

Respondents who had a history of mental illness had poorer mental health outcomes [38]. The results of our investigation lend support to this study's findings, showing that those with a history of mental illness had a 4.31 times higher prevalence of depressive symptoms than those without a history. Notably, the injury was also a significant determinant factor for greater depression symptoms. It has been regularly related to poor mental health around the world [39]. According to our data, the probabilities of developing depressive symptoms were 1.823 times higher in persons who had been injured than in those who had not. These findings are congruent with those of other postwar mental health surveys.

Cigarette smoking was found to be statistically insignificant in the current study. However, according to other study findings, smoking status was the most significant predictor of developing significant depressive symptoms [40]. According to the study's findings, CES-D scores for current smokers with nicotine dependence symptoms were significantly higher than those of non-smokers [41].

### 4.1 Strengths and weaknesses of the study

This study attempted to assess the depression symptoms among people of the south Wollo zone following the liberation of their area from the TPLF Led Force. There are some limitations to this study. (i) In this cross-sectional study, we are unable to establish a causal relationship. (ii) There might be other factors, in addition to those we considered, that were linked to the prevalence of depressive symptoms among residents and need further investigation. Despite this flaw, this study is one of the very few in developing countries to use standardized procedures and in-depth analysis.

## 5 Conclusions

The key finding of this study was that after being freed from the TPLF-led army, locals reported a higher prevalence of depression symptoms. Depressive symptoms were more likely to occur in those who were being alcoholics, chewed Khat, had a history of mental illness, destroyed property, who had lack of food or water, and were ill without receiving medical attention. Coping strategies for reducing depressive symptoms caused by invasion could include not drinking too much alcohol, not chewing khat, delivering necessities to allow them to eat a healthy diet and gain access to water, providing consultation to those with a history of mental illness, and assisting those whose property has been destroyed. To ensure the mental health of inhabitants, interventions should be carried out.

## Declarations

### Ethics approval and consent to participate

The research review committee of the College of Natural and Computational Science at Mizan-Tepi University granted ethical clearance. The participants were informed of the study's goal. Verbal informed consent was obtained from each participant because the participants included all of the locals, including those who were illiterate and reluctant to sign or place their thumbprints on consent papers. The Mizan-Tepi University research and ethics review board waived the need for study participants to give verbal and written informed consent, however, the data were kept anonymous and secret. This study was conducted per the Declaration of Helsinki.

### Author contribution statement

Seid Ali Tareke: Conceived and designed the experiments; Performed the experiments; Analyzed and interpreted the data; Contributed reagents, materials, analysis tools or data; Wrote the paper.

Mesfin Esayas Lelisho: Analyzed and interpreted the data; Contributed reagents, materials, analysis tools or data; Wrote the paper.

Abdu Hailu, Mohamednur Kadire, Yasin Negash, Kibrealem Sisay, Fikadu Zawdie, Ebrahim Chaine, Shukure Uomer: Contributed reagents, materials, analysis tools or data; Wrote the paper.

### Funding statement

This research did not receive any specific grant from funding agencies in the public, commercial, or not-for-profit sectors.

### Data availability statement

Data will be made available on request.

### Declaration of interest's statement

The authors declare that there are no competing interests.
